# Deep Learning-Based MRI Analysis Reveals Lewy Body Co-Pathology Accelerates Brain Aging in Alzheimer’s Disease

**DOI:** 10.21203/rs.3.rs-6874970/v1

**Published:** 2025-06-26

**Authors:** Babak Ahmadi, Melissa Armstrong, Breton M. Asken, Mostafa Reisi-Gahrooei, Abbas Babajani-Feremi, the Alzheimer’s Disease Neuroimaging Initiative, the Australian Imaging Biomarkers and Lifestyle flagship study of ageing

**Affiliations:** 1 Department of Industrial and Systems Engineering, University of Florida, Gainesville, FL, USA.; 2 Department of Neurology, University of Florida, Gainesville, FL, USA.; 3 Magnetoencephalography (MEG) Lab, The Norman Fixel Institute of Neurological Diseases, University of Florida Health, Gainesville, FL, USA.; 4 Department of Clinical and Health Psychology, University of Florida, Gainesville, FL, USA.

**Keywords:** Alzheimer’s disease, Lewy body, Co-pathology, Brain age, Deep learning, MRI

## Abstract

Alzheimer’s disease (AD) and Lewy body (LB) pathology frequently co-occur. Recent advances in cerebrospinal fluid (CSF) α-synuclein seed amplification assays (SAA) enable *in vivo* detection of LB pathology, offering new opportunities to elucidate its combined effects with AD on neurodegeneration. We trained a deep learning model on multi-cohort MRI scans from 4,355 cognitively unimpaired individuals to estimate brain age and applied it to 803 cognitively impaired participants, who were classified into four AD/LB pathology subgroups using the p-tau_181_/Aβ_42_ ratio to specify AD pathology and SAA status to determine LB pathology. The co-pathology subgroup (AD+LB+) exhibited the most accelerated brain aging, with saliency maps revealing more pronounced neurodegeneration, aligning with its steeper longitudinal atrophy in various neuroanatomical regions and corresponding cognitive deficits. These findings underscore LB pathology’s synergistic role in amplifying AD-related neurodegeneration, highlighting the importance of combined biomarker assays and targeted interventions for individuals harboring co-existing AD and LB pathology.

## 1. Introduction

Alzheimer’s disease (AD) and Lewy body (LB) disease are the most prevalent neurodegenerative disorders affecting the aging population^1^. AD is characterized by the accumulation of amyloid-beta (Aβ) plaques and tau neurofibrillary tangles, leading to neurodegeneration and progressive cognitive decline^2^. LB disease, which presents as the clinical syndromes of Parkinson’s disease (PD) and dementia with Lewy bodies (DLB), is characterized by intraneuronal aggregation of misfolded α-synuclein (α-syn) proteins that form Lewy bodies and Lewy neurites^3,4^. While α-syn accumulation is the hallmark of LB diseases, it is also a common co-pathology in patients with AD^2,5,6^. Post-mortem studies have shown that approximately 30–50% of autopsy-confirmed AD cases exhibit comorbid α-syn pathology in addition to amyloid-beta (Aβ) and tau depositions^7–10^. This overlap suggests a synergistic interaction that potentially accelerates both neurodegeneration and cognitive decline beyond what is observed in either condition alone^2,5,11–14^, thereby increasing diagnostic and therapeutic complexity. Accurately detecting and characterizing co-pathologies is therefore critical for improving prognosis and guiding more targeted therapeutic strategies.

Several well-validated biomarkers of AD pathology are now routinely used in research and increasingly in clinical care, including cerebrospinal fluid (CSF) biomarkers, Aβ positron emission tomography (PET), and emerging blood-based assays^15^. Recent advancements in CSF biomarker techniques, particularly the α-syn seed amplification assay (SAA), have enhanced the *in vivo* detection of LB pathology^16–18^. Across diverse clinical^19,20^ and neuropathological^2,5,21–23^ cohorts, SAA demonstrated high specificity and sensitivity, underscoring its utility in providing reliable diagnostic insights into LB pathology, offering a vital tool for understanding the role of LB pathology across neurodegenerative diseases.

Building on these advancements, SAA positivity has been linked to earlier symptom onset^24^, faster cognitive decline and greater cortical hypometabolism^2^ (especially when co-existing with AD pathology), and structural atrophy^3^ (e.g., in the nucleus basalis of Meynert). Despite these insights, most studies have focused on isolated dimensions of pathology, such as biomarker positivity or structural atrophy, without integrating them into a longitudinal multimodal framework. This gap underscores the need for more comprehensive, multimodal methodologies that can illuminate how LB co-pathology exacerbates AD-related clinical deterioration and neuronal loss, beyond either pathology alone.

In parallel with the expanding clinical applications of α-syn SAA and the insights they provide into LB pathology, significant advances in deep learning (DL) have opened new avenues in neurodegenerative research, including its integration with neuroimaging^25–31^, such as MRI. Structural MRI studies have shown that increased age and the existence of neurodegenerative diseases are associated with structural alterations, including reductions in gray matter volume within the hippocampus and insular cortex^32–35^. Such findings have motivated the deployment of DL algorithms on MRI data to develop potent biomarkers for neurodegeneration. Chief among these biomarkers is the brain age gap (BAG)—the difference between a DL–predicted brain age (BA) and an individual’s chronological age (CA)^28,36–39^. Notably, multiple studies have demonstrated robust performance of BA prediction models based on T1-weighted MRI and associated the greater brain age gaps to worse clinical outcomes^25,29,40–43^, underscoring the utility of BAG as a measure of neurodegenerative burden.

Prior studies have consistently shown that individuals with AD or clinically diagnosed LB disease often exhibit elevated BAGs relative to cognitively unimpaired individuals, reflecting deviations from normative aging^25,26,36^. However, these studies have largely focused on isolated neurodegenerative diseases and, in the case of LB disease, have relied on clinical criteria (e.g., consensus guidelines for DLB^44^) rather than detection of misfolded α-synuclein. Consequently, no work to date has investigated whether *in vivo* LB pathology contributes to BAGs or, more critically, whether its combination with AD pathology further accelerates brain aging and neurodegeneration beyond the effects of AD alone, leaving its impact unclear. This gap in the literature is particularly notable since LB pathology often contributes to distinct patterns of structural decline—above and beyond the hallmark atrophy seen in AD^26^. Prior neuropathology and MRI-based investigations of α-syn–associated atrophy have also shown inconsistent findings regarding its overlap with AD-related regions (e.g., the medial temporal lobe)^3,45–48^. Equally missing is an MRI-based longitudinal perspective that evaluates how LB pathology might compound over time, particularly against the backdrop of AD, and how it ultimately influences region-specific and overall neurodegeneration, with downstream effects on cognition. Therefore, there is a critical need for multifaceted, longitudinal analyses to disentangle how LB pathology interacts with AD to compound neurodegeneration.

**Figure 1.**
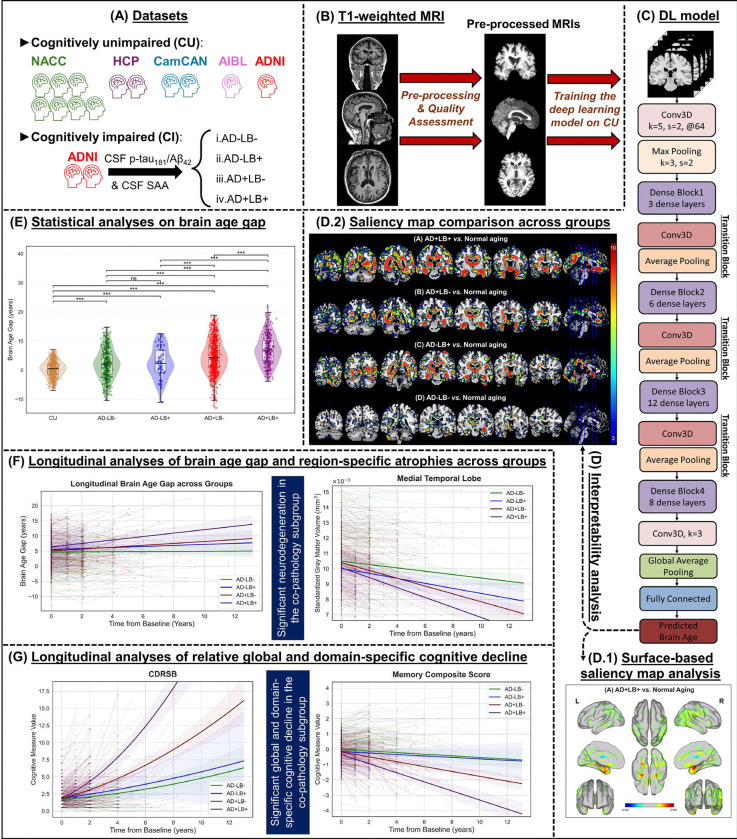
An overview of the study design and analytic workflow, labeled (A)–(G). **(A)** T1-weighted MRI scans were collected from five major cohorts (NACC, HCP, CamCAN, AIBL, and ADNI; each symbol represents ~350 participants), providing cognitively unimpaired (CU) scans for training the deep learning (DL) model, plus cognitively impaired participants with biomarker-defined Alzheimer’s disease (AD) and Lewy body (LB) pathology. **(B)** All MRI scans underwent standardized preprocessing (FreeSurfer, affine registration) and rigorous quality assessments to prepare the data for the DL model. **(C)** A 3D-DenseNet architecture was trained on CU scans and was used to predict brain age in all pathology subgroups. **(D)** After training the model, interpretability analyses were conducted: **(D1)**, **(D2)** Saliency maps were computed for AD/LB subgroups versus the normative aging reference, highlighting regions driving the higher brain age gaps for the AD/LB pathology subgroups. **(E)** Comparisons of brain age gaps quantified deviations from normal aging across AD/LB subgroups. **(F)** Longitudinal trajectories of brain age gap and structural measures were assessed, capturing progressive atrophy. **(G)** Complementary analyses of global and domain-specific cognition linked structural findings to clinical decline. MRI: Magnetic resonance imaging; NACC: National Alzheimer’s Coordinating Center; HCP-A: Human Connectome Project in Aging; CamCAN: Cambridge Centre for Ageing and Neuroscience; AIBL: Australian Imaging, Biomarkers & Lifestyle; ADNI: Alzheimer’s Disease Neuroimaging Initiative; CU: Cognitively unimpaired; AD: Alzheimer’s disease; LB: Lewy body; DL: Deep learning; CI: Cognitively impaired; CDRSB: Clinical dementia rating, sum of boxes

In the present study, first, we established an accurate DL–based brain age estimation model, which was validated across multiple cohorts and could robustly capture normal aging trajectories. Second, using validated *in vivo* biomarkers of Aβ, tau and α-syn pathology, we examined how AD, LB, and the combined AD+LB pathologies drove elevated brain ages that deviated from typical aging. We further conducted sex-wise analyses to investigate sex vulnerability in AD and AD+LB co-pathology. Third, we employed interpretability analyses of the DL model-driven saliency maps in AD/LB pathology subgroups relative to the normal aging reference group, which allowed us to pinpoint the regions most vulnerable to LB co-pathology on an AD background. Fourth, we assessed the longitudinal trajectories of BAGs and region-specific volumetric changes to clarify how LB co-pathology modifies the spatial-temporal patterns of brain atrophy. This approach aimed to explore how coexistent LB pathology intensifies the typical patterns of AD-related neurodegeneration, while mapping concurrent declines in global and domain-specific cognition, thereby elucidating a mechanistic link between the structural footprint of co-pathology and accelerated clinical deterioration. An overview of the study design is presented in Figure 1.

## 2. Results

### 2.1. Cohort characteristics

The cognitively unimpaired (CU) dataset was drawn from five cohorts—National Alzheimer’s Coordinating Center (NACC), Alzheimer’s Disease Neuroimaging Initiative (ADNI), Australian Imaging, Biomarkers & Lifestyle (AIBL)^49^, Cambridge Centre for Ageing and Neuroscience^50,51^ (CamCAN), and Human Connectome Project in Aging (HCP-A)—and restricted to one scan per participant to minimize data leakage and the risk of overfitting. We first combined these data (*n* = 4,355; 65.9 ± 13.3 years, 63.2% female) and as described in Methods, partitioned them into training, validation, and test sets, carefully stratifying by chronological age. A one-way analysis of variance (ANOVA) showed no significant difference in age distributions among these subsets (*F* = 0.007, *p* > 0.05), and a chi-square test confirmed no significant difference in sex (*χ*^2^ = 1.29, *p* > 0.05). **Supplementary Table 1** summarizes these characteristics of the CU individuals for training the DL model.

Among all 803 cognitively impaired (CI) individuals with AD/LB pathology, 195 fell into the AD−LB− category, 46 into the AD−LB+, 396 into the AD+LB−, and 166 into the AD+LB+. The AD−LB− subgroup was significantly younger than the three biomarker-positive subgroups (*F* = 4.6, *p* < 0.01), although no significant differences were found among the three biomarker-positive groups themselves. The AD−LB+ subgroup had notably fewer female participants than the others (28.3% *vs*. 39.2–42.4%). Table 1 summarizes demographic and clinical features for these subgroups.

### 2.2. Brain age estimation in cognitively unimpaired individuals

**Figure 2.**
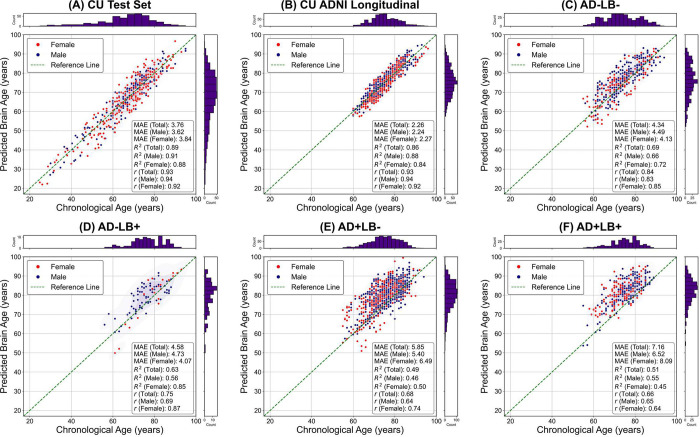
Model performance in predicting brain age across cognitively unimpaired and cognitively impaired AD/LB subgroups. Scatter plots compare predicted brain age (y-axis) and chronological age (x-axis) for **(A)** cognitively unimpaired individuals, highlighting the combined multicohort test set (CU Test Set), **(B)** a separate longitudinal subset from ADNI (CN ADNI Longitudinal), **(C)**-**(F)** each AD/LB pathological subgroup. Each datapoint represents a scan, colored by sex (red dots for females, blue dots for males).. The diagonal dashed line shows a perfect 1:1 correspondence between predicted and true ages. Histograms along the axes depict the distribution of chronological ages and predicted brain ages for each subset. Mean absolute error (MAE), coefficient of determination (*R*^2^), and Spearman’s correlation (*r*) are reported for each group (and by sex) in the inset boxes, underscoring robust model generalization in CU individuals and elevated brain age gaps in pathological subgroups. CU: Cognitively unimpaired; AD: Alzheimer’s disease; LB: Lewy body; ADNI: Alzheimer’s Disease Neuroimaging Initiative.

We trained a 3D-DenseNet model to estimate BA from T1-weighted MRI scans of CU individuals, encompassing a broad age range (23–100 years), ensuring robust capture of normative aging trajectories. After training, we evaluated the model on the CU test set (*n*_test_ = 436), which included scans from all five cohorts. This diverse test set assessed the model’s ability to generalize across site- and population-level differences. The bias corrected BAG averaged near zero (−0.1 ± 0.22 years, mean absolute error [MAE] = 3.76 ± 0.13 years), with strong agreement (*R*^*2*^ = 0.89) between predicted and chronological ages (Figure 2A).

Next, we applied the model to the longitudinal scans of the CU individuals in ADNI. This step was aimed to test the model’s reliability in capturing normal aging in this cohort and to generate a reference point for subsequent comparisons with ADNI-derived AD/LB pathology subgroups. After bias correction, the average BAG was 0.31 ± 0.11 years and the MAE was 2.26 ± 0.06 years, suggesting robust performance on 691 scans of the CU data in ADNI (Figure 2B).

### 2.3. Brain age estimation in pathology subgroups

**Figure 3.**
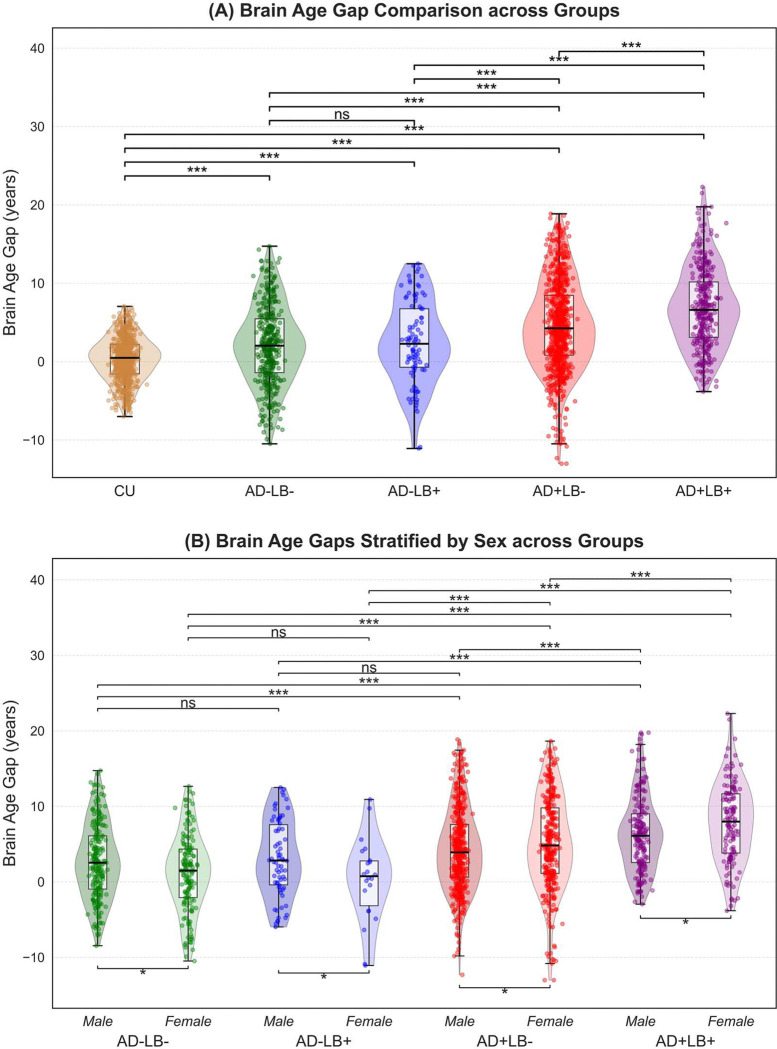
Bias-corrected brain age gaps across subgroups. **(A)** Violin plots showing the distribution of bias-corrected brain age gaps across the four pathological subgroups (AD−LB−, AD−LB+, AD+LB−, and AD+LB+), as well as the cognitively unimpaired reference (CU). The black box in each violin denotes the interquartile range and median, while whiskers mark the minimum and maximum data points within 1.5 × IQR. Post hoc analyses indicate that all pathological subgroups differ significantly from CU (***p < 0.001), with the co-pathology subgroup (AD+LB+) showing the largest deviation. These results underscore the compounded neurodegenerative impact when AD and LB processes co-occur. **(B)** Brain age gaps stratified by sex within each AD/LB pathological subgroup. Violin plots display the distribution of age gap estimates, with box plots indicating the median and interquartile ranges. Pairwise *t*-tests (Holm–Šídák corrected) reveal that in the absence of AD (AD−LB− or AD−LB+), males show significantly higher brain age gaps, whereas in AD+ subgroups (AD+LB−, AD+LB+), females exhibit higher brain age gaps. This pattern underscores potential sex-specific susceptibility once AD pathology is present. One-way ANOVAs conducted separately for males and females confirm that co-pathology (AD+LB+) yields the largest deviation in both sexes, suggesting that LB co-pathology can intensify sex-specific vulnerability. (*ns*: not significant; *p < 0.05; **p < 0.01; ***p < 0.001). AD: Alzheimer’s disease; LB: Lewy body; IQR: Interquartile range; ANOVA: Analysis of variance.

Deploying the model trained with normative cohorts, we estimated BA in the four CI AD/LB pathology subgroups. Figure 3 shows the distribution of these bias-corrected brain age gaps, and corresponding summary results are presented in **Supplementary Table 2**. All AD/LB pathology subgroups showed significantly elevated brain age gaps relative to the CU reference—indicating that their structural MRI-derived brain ages exceeded chronological ages to a greater extent than in CU individuals. Notably, as shown in Figures 2C–F, the co-pathology subgroup (AD+LB+) exhibited the largest deviation (6.93 ± 0.29 years; MAE = 7.16 ± 0.27), exceeding that of both AD+LB− (4.64 ± 0.20 years; MAE = 5.85 ± 0.16) and AD−LB+ (2.40 ± 0.55 years; MAE = 4.58 ± 0.37), as well as AD−LB− (2.08 ± 0.26 years; MAE = 4.34 ± 0.17).

A one-way ANOVA indicated a highly significant group effect on brain age gaps (*F* = 136.06, *p* < 10^−103^). Post hoc Holm–Šídák tests further indicated that each pathology subgroup differed significantly from the CU reference (*p* < 2×10^−8^), confirming markedly elevated brain age gaps across all AD/LB subgroups. As depicted in Figure 3A, among the CI pathology subgroups themselves, the only nonsignificant pairwise comparison was between AD−LB− and AD−LB+. Further subgroup comparisons revealed that the co-pathology condition (AD+LB+) surpassed both AD or LB pathology alone (*p* < 0.001), underscoring a potential synergistic effect on structural brain aging when AD and LB co-occur.

### 2.4. Sex differences in brain age gaps

To investigate the influence of sex on brain age gaps across the pathology subgroups, we conducted two complementary analyses. First, we performed pairwise comparisons of males versus females within each pathological subgroup using two-sample *t*-tests. Second, we assessed differences among subgroups for each sex separately using one-way ANOVAs. Figure 3B displays the distribution of the brain age gaps for males and females in each pathology subgroup.

From the within-group comparisons, in the AD− subgroups (AD−LB− and AD−LB+), males showed significantly higher brain age gaps than females (*p* < 0.05), whereas in the AD+ subgroups (AD+LB− and AD+LB+), females exhibited significantly higher brain age gaps than males (*p* < 0.05). This reversal from lower to higher apparent BA in females suggests that sex-specific factors may differentially modulate susceptibility once AD pathology is present.

When pooling all males and all females separately by pathological subgroup, one-way ANOVAs indicated that both sexes had significantly elevated brain age gaps in AD+LB+ relative to all other groups, mirroring the pattern seen in the combined analysis. The difference between AD−LB− and AD−LB+ was not significant in either sex, paralleling the group-level results.

### 2.5. Interpretability of brain age prediction model

**Extended Data Figure 1.**
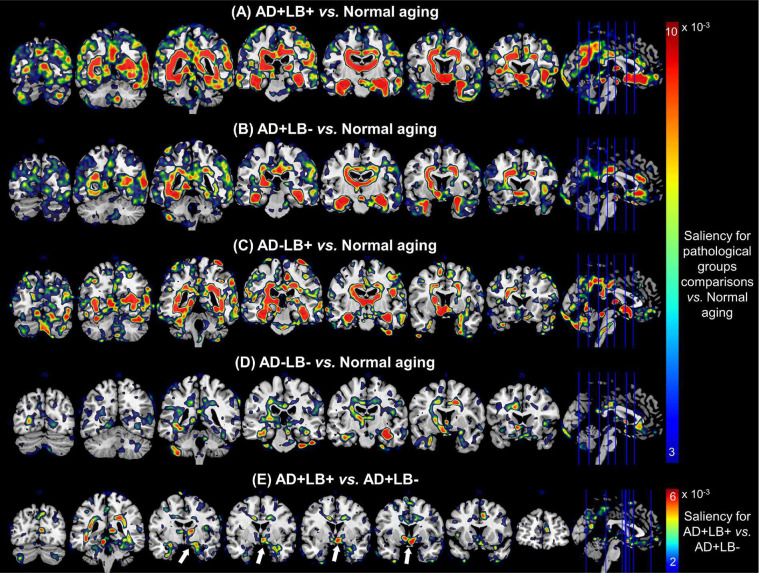
Saliency map comparisons for AD/LB subgroups *vs.* Normal Aging and AD+LB+ *vs.* AD+LB−. **(A)** AD+LB+ *vs*. Normal Aging, **(B)** AD+LB− *vs*. Normal Aging, **(C)** AD−LB+ *vs*. Normal Aging, **(D)** AD−LB− *vs*. Normal Aging, and **(E)** AD+LB+ *vs*. AD+LB−. Saliency maps are overlaid on the standart MNI template, and various slices of the coronal view are presented. More pronoucned saliency is observed for the co-pathology subgroup *vs.* normal aging referece, compared to other subgroups *vs.* this reference group. Comparing AD+LB+ *vs.* AD+LB−, we can observe a positive saliency cluster near the cholinergic basal forebrain (indicated by white arrows), including the Nucleus Basalis of Meynert (NBM), highlighting heightened relevance of these nuclei in distinguishing co-pathology from AD alone.

**Figure 4.**
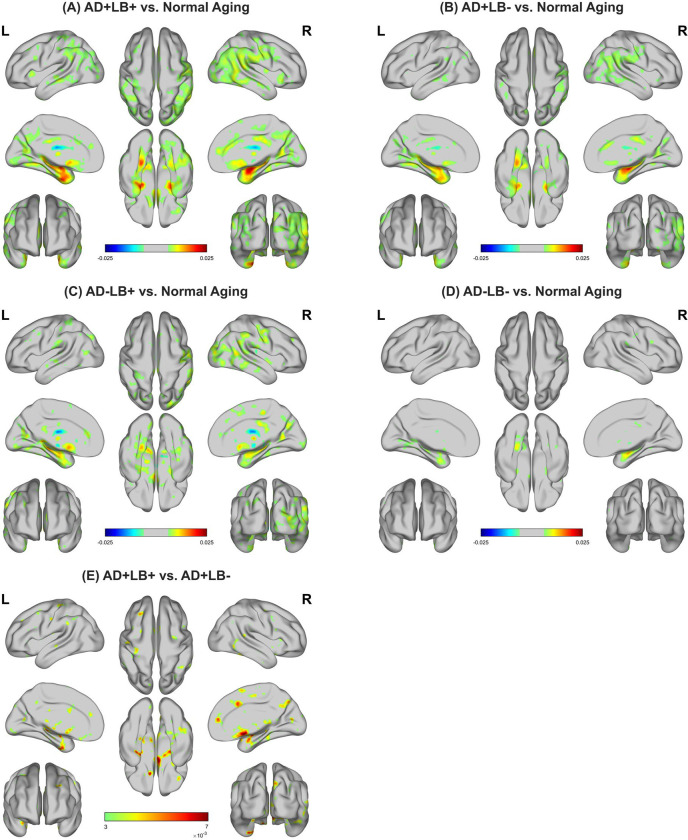
Group-level difference saliency maps, driven from the deep learning model, highlighting regions contributing to elevated brain age in AD/LB subgroups. **(A–D)** The difference between the averaged saliency for each AD/LB subgroup versus the cognitively unimpaired (CU) reference; warmer colors (yellow–red) indicate stronger contributions to the model’s higher brain age predictions in the pathological subgroup. Conversely, cooler colors (blue–cyan) mark areas in which saliency is greater for the cognitively unimpaired cohort. The AD+LB+ demonstrates a more extensive involvement than AD+LB− or AD−LB+, consistent with an amplified neurodegeneration in co-pathology. **(E)** Direct comparison of AD+LB+ vs. AD+LB−, isolating the additional effect of LB on an AD background. Prominent saliencies involve cholinergic basal forebrain, and parts of medial temporal structures, right cingulum, right precuneus, and left fusiform gyrus consistent with a synergistic neurodegenerative footprint in co-pathology. AD: Alzheimer’s disease; LB: Lewy body.

To gain insight into the anatomical regions most relevant to our BA predictions from the MRI scans, we generated saliency maps for each individual and averaged them over each pathology subgroup. These were then compared to the averaged saliency map derived from CU individuals—representative of normative aging. This approach aimed to account for normal aging effects and highlight the specific regions driving higher brain age gaps, thereby identifying areas associated with neurodegenerative changes in the AD/LB pathology subgroups. As illustrated in Figure 4A–D (and Extended Data Figure 1A–D), these difference maps revealed that the co-pathology subgroup (AD+LB+) had comparatively heightened saliency across the medial surfaces, including the medial temporal lobe (MTL) and related subcortical structures. Specifically, the AD+ subgroups showed clear evidence of intensified atrophy in the amygdala, hippocampus, fusiform gyrus, parahippocampal, and entorhinal cortex, with AD+LB+ demonstrating wider involvement in these regions than AD+LB−. We further observed slightly elevated saliency in parts of the right cingulum, cuneus, precuneus, and lingual for AD+LB+ relative to AD+LB−. Additionally, the LB+ subgroups showed relatively greater saliency in the occipital cortex, mid-temporal (posterior aspect), and calcarine cortex compared to LB− subgroups, along with elevated involvement of the thalamus, caudate, and olfactory areas. Pairwise comparisons of mean saliency values using the AAL atlas are provided in **Supplementary Tables 3–6**.

Moreover, we compared the averaged saliency map of AD+LB+ directly against that of AD+LB− to isolate the contribution of LB pathology on an existing AD background. As shown in Figure 4E (and Extended Data Figure 1E), a pronounced positive saliency cluster appeared near the cholinergic basal forebrain, including the nucleus basalis of Meynert (NBM), as well as in parts of the MTL, right cingulum, right precuneus, and left fusiform gyrus, highlighting their vulnerability to LB pathology.

### 2.6. Longitudinal dynamics of brain aging

To further elucidate how LB co-pathology exacerbates AD processes, we evaluated brain aging from a longitudinal perspective. Specifically, we fitted linear mixed models (LMMs) to the 1,574 longitudinal scans from all participants across the four AD/LB subgroups, while baseline comparisons were also analyzed via a general linear model, adjusting for baseline age, sex, cognitive state, and level of education, with AD−LB− as the reference group. Model comparisons via Bayesian Information Criterion (BIC) favored a linear model over a quadratic one.

**Figure 5.**
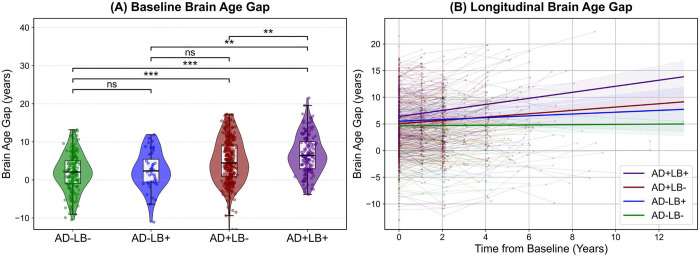
Baseline and longitudinal comparisons of brain age gaps across pathology subgroups. Violin plots in **(A)** illustrate the distribution of brain age gaps at baseline across the four AD/LB subgroups, with overlaid boxplots indicating median and interquartile ranges. Pairwise group comparisons were conducted using a generalized linear model (*ns*: not significant; *p < 0.05; **p < 0.01; ***p < 0.001). In **(B)**, thin lines represent individual changes in brain age gap over time, while thick lines with shaded bands indicate group-level linear fits and corresponding 95% confidence intervals. The AD+LB+ subgroup exhibits the steepest slope, reflecting a significantly accelerated divergence from AD−LB− (*p* < 0.001), AD−LB+ (*p* < 0.01), and AD+LB− (*p* < 0.001), highlighting the additional neurodegenerative burden imposed by LB co-pathology in the presence of AD. All models are adjusted for baseline age, sex, cognitive status, and education level, with multiple comparison corrections applied. AD: Alzheimer’s disease; LB: Lewy body.

At baseline, relative to AD−LB−, both AD+LB− (*β* = 2.25, SE = 0.46, *p* < 0.0001) and AD+LB+ (*β* = 3.53, SE = 0.56, *p* < 0.0001) displayed significantly higher brain age gaps. Pairwise contrasts further indicated that AD+LB+ also exceeded AD−LB+ (*β* = 2.59, SE = 0.85, *p* < 0.01) and AD+LB− (*β* = 1.28, SE = 0.47, *p* < 0.01) at baseline (Figure 5A). Turning to slopes (i.e., the rate of change in BAG per year), relative to AD−LB−, which had a negligible yearly slope (*β* = 0.03), AD+LB+ (*β* = 0.54, SE = 0.14, *p* < 0.001) and AD+LB− (*β* = 0.29, SE = 0.10, *p* < 0.01) showed significant increases over time. In AD−LB+ (*β* = 0.14, SE = 0.18, *p* > 0.41), the slope was numerically higher but did not reach statistical significance. Pairwise contrasts confirmed that AD+LB+ outpaced AD+LB− (*β* = 0.25, *p* < 0.001) and AD−LB+ (*β* = 0.40, *p* < 0.01), underscoring the additional neurodegenerative burden imposed by co-occurring AD and LB pathologies (Figure 5B). Overall, these findings indicate that although AD positivity alone accelerates brain aging, the concomitant presence of LB pathology amplifies this effect even further, leading to a more pronounced and rapidly evolving deviation from normative aging trajectories.

### 2.7. Longitudinal effect of co-pathology on region-specific atrophy

**Extended Data Figure 2.**
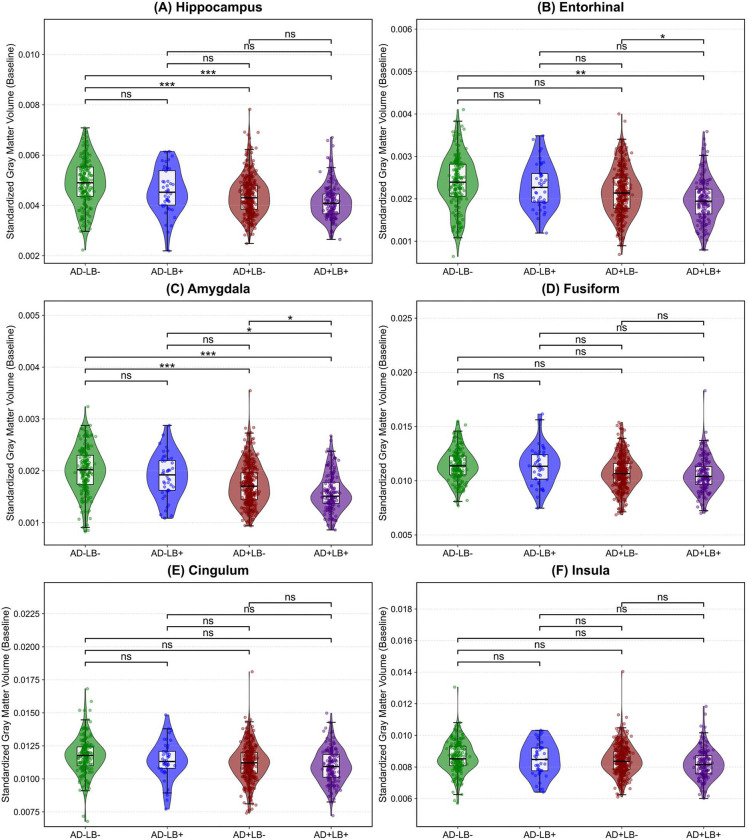
Baseline volumetric comparisons across AD/LB pathology subgroups. Violin plots illustrate the distribution of standardized gray matter volume at baseline across four AD/LB pathological subgroups for **(A)** Hippocampus, **(B)** Entorhinal cortex, **(C)** Amygdala, **(D)** Fusiform gyrus, **(E)** Cingulum, and **(F)** Insular cortex, with overlaid boxplots indicating median and interquartile ranges. The top bars and corresponding significance labels reflect pairwise group comparisons under a generalized linear model adjusted for baseline age, sex, cognitive state, and level of education, with p-values corrected for multiple comparisons. (*ns*: not significant; *p < 0.05; **p < 0.01; ***p < 0.001).

**Extended Data Figure 3.**
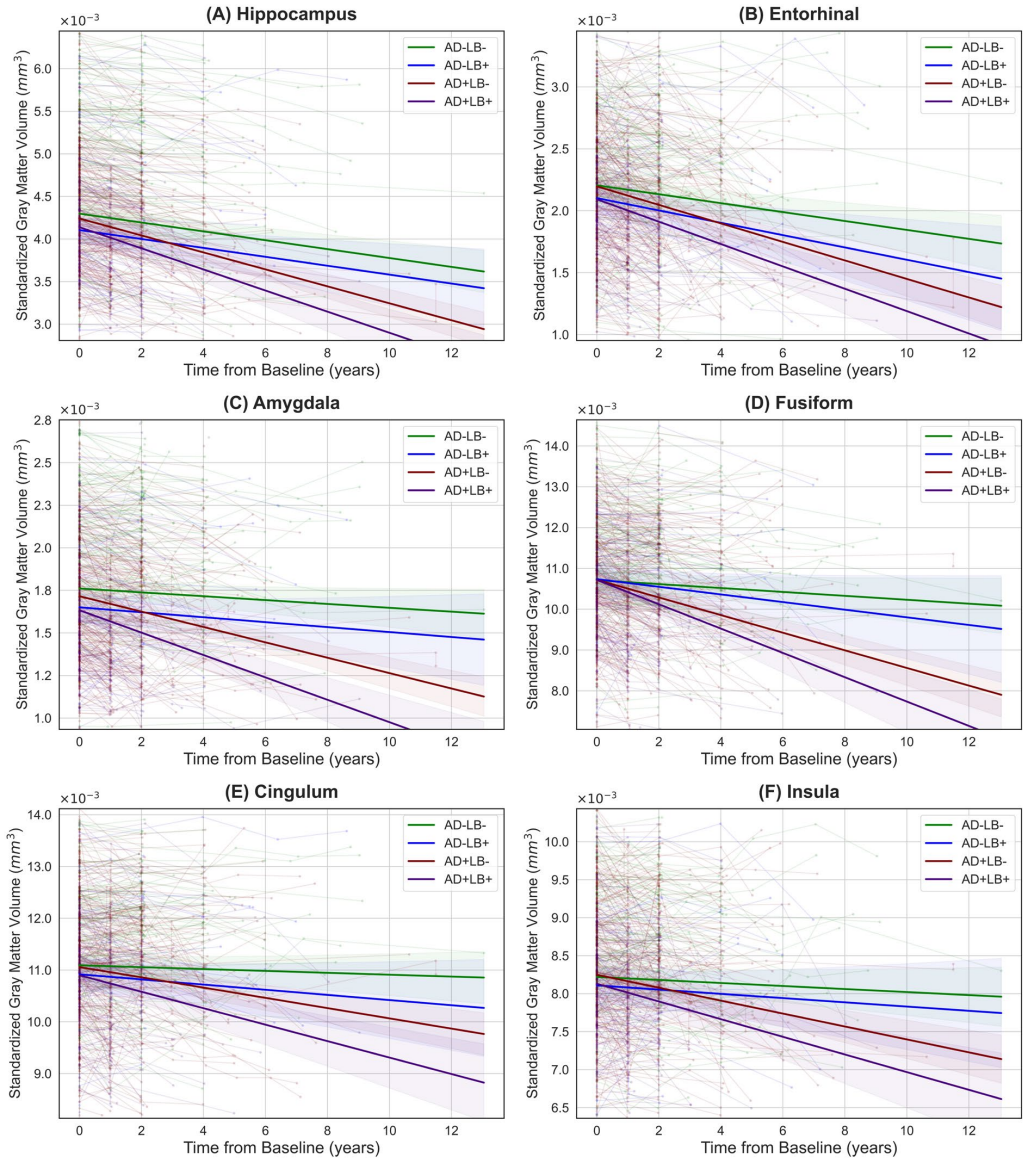
Extended longitudinal analyses of volumetric atrophy. Each panel shows normalized volumes (y-axis) over time (x-axis) for **(A)** Hippocampus, **(B)** Entorhinal, **(C)** Amygdala, **(D)** Fusiform, **(E)** Cingulum, and **(F)** Insular cortex across the four AD/LB pathological subgroups. Each thin line represents an individual’s change in normalized volume over time from baseline, while the thicker lines with shaded bands depict group-level linear fits and their corresponding 95% confidence intervals. In all regions, AD+LB+ demonstrates the steepest decline relative to AD+LB−, AD−LB+, and AD−LB− (*p* < 0.0001–0.05 across contrasts), consistent with a faster and more pronounced atrophy pattern under LB co-pathology. All models are corrected for baseline age, sex, cognitive state, and level of education. Also, all *p*-values are adjusted for multiple comparison.

**Figure 6.**
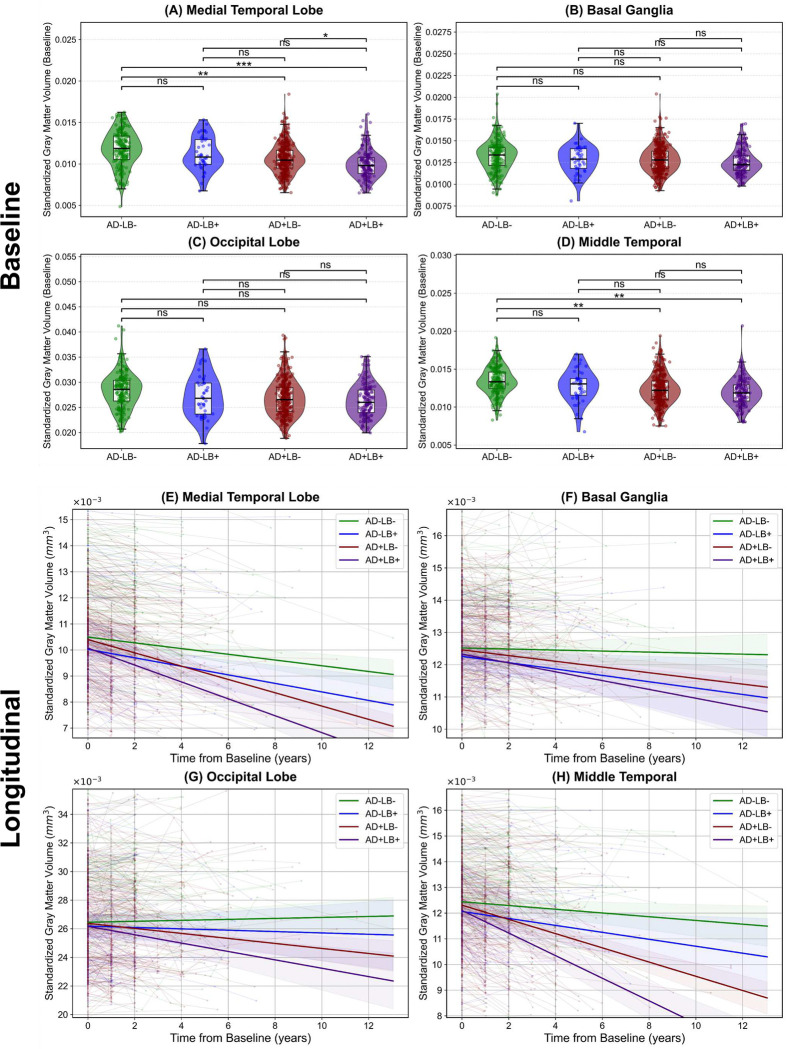
Baseline and longitudinal comparisons of gray matter volume in key AD and LB-related regions across AD/LB pathology subgroups. **(A)–(D)** panels illustrate the distribution of standardized gray matter volume at baseline for the **(A)** medial temporal lobe, **(B)** basal ganglia, **(C)** occipital lobe, and **(D)** middle temporal cortex across four AD/LB pathological subgroups. Overlaid boxplots indicate medians and interquartile ranges. The top bars and corresponding significance labels reflect pairwise group comparisons under a generalized linear model (*ns*: not significant; *p < 0.05; **p < 0.01; ***p < 0.001). **(E)–(H)** panels depict the longitudinal trajectories of standardized gray matter volume over time for the same four regions. Individual trajectories are shown as thin lines, while thick lines with shaded bands represent group-level linear fits and their corresponding 95% confidence intervals. Relative to the single-pathology subgroups (AD−LB+ or AD+LB−) and AD−LB− reference, the co-pathology subgroup (AD+LB+) consistently demonstrates the fastest volumetric declines. For instance, in the MTL, AD+LB+ exhibits significantly steeper atrophy than AD−LB− (*p* < 0.0001) and AD+LB− (*p* < 0.001); in the occipital lobe, AD+LB+ exceeds AD−LB− (*p* < 0.001) and AD+LB− (*p* < 0.05); in the basal ganglia, AD+LB+ differs from AD−LB− (*p* < 0.05); and in the middle temporal cortex, AD+LB+ surpasses AD−LB− and AD+LB− (*p* < 0.0001), indicating a more pronounced atrophy pattern under LB co-pathology. All models are corrected for baseline age, sex, cognitive state, and level of education. Also, all *p*-values are adjusted for multiple comparison. AD: Alzheimer’s disease; LB: Lewy body.

We investigated specific neuroanatomical regions to gain a more granular understanding and clarify how these proteinopathies combine to drive atrophy beyond what either pathology alone might incur. Informed by our saliency map findings and prior studies, we focused on the gray matter volume of the MTL—aggregating hippocampus, amygdala, entorhinal cortex, and parahippocampal gyrus—and key regions outside the MTL, including the basal ganglia, occipital lobe, and middle temporal cortex. Figure 6 shows representative baseline and longitudinal comparisons across pathology subgroups, with additional results for the fusiform, cingulum, insula, and MTL sub-regions provided in Extended Data Figures 2, 3, with more details available in the Supplementary Materials. Aligned with our global BA analyses, BIC comparisons consistently supported linear rather than quadratic trajectories in each ROI.

At baseline, in the MTL, both AD+LB− (*β* = −5.01 × 10^−4^, SE = 1.52 × 10^−4^, *p* < 0.01) and AD+LB+ (*β* = −8.52 × 10^−4^, SE = 1.84 × 10^−4^, *p* < 0.0001) showed significantly lower volumes compared to AD−LB−, indicating pronounced baseline atrophy. AD−LB+ did not reach statistical significance. The baseline volume of MTL in AD+LB+ was also significantly lower than that of AD+LB− (*β* = −3.51 × 10^−4^, SE = 1.52 × 10^−4^, *p* < 0.05). In the basal ganglia and occipital lobe, none of the subgroups significantly diverged from AD−LB− or from each other. For the middle temporal cortex, AD+LB+ (*β* = −6.60 × 10^−4^, SE = 2.01 × 10^−4^, *p* < 0.01), AD+LB− (*β* = −5.00 × 10^−4^, SE = 1.62 × 10^−4^, *p* < 0.01) and AD−LB+ fell below AD−LB−, while AD−LB+ did not reach statistical significance. Additionally, pairwise contrasts among AD+LB+, AD+LB−, and AD−LB+ were nonsignificant at baseline.

Over time, compared to AD−LB−, AD+LB− (*β* = −1.45 × 10^−4^, *p* < 0.0001) and AD+LB+ (*β* = −2.14 × 10^−4^, *p* < 0.0001) exhibited steeper MTL atrophy slopes, with AD+LB+ being the highest (*vs.* AD+LB−: *β* = −6.90 × 10^−5^, *p* < 0.001), aligning with the increased saliency observed for MTL regions, indicating that co-occurring LB pathology amplifies existing AD-related neurodegeneration within these highly vulnerable limbic areas. In the basal ganglia, AD+LB− (*β* = −7.30 × 10^−5^, *p* < 0.05) and AD+LB+ (*β* = −1.21 × 10^−4^, *p* < 0.05) declined faster than AD−LB−, although their difference remained nonsignificant (uncorrected *p* = 0.036, adjusted *p* ≈ 0.073). The occipital lobe again showed the most pronounced slope in AD+LB+ (*β* = −3.27 × 10^−4^, *p* < 0.001), with AD+LB− also accelerated (*β* = −2.09 × 10^−4^, *p* < 0.001). AD−LB+ did not reach the threshold for significance. Pairwise comparisons further confirmed AD+LB+ surpassed AD+LB− (*p* < 0.05), reinforcing that concomitant LB pathology intensifies atrophy in visually related cortices beyond what is observed in AD alone. Finally, in the middle temporal cortex, AD+LB+ exceeded AD+LB− (*β* = −1.63 × 10^−4^, *p* < 0.0001), whereas AD+LB− itself exhibited steeper slopes compared to AD−LB− (*β* = −2.04 × 10^−4^, *p* < 0.0001).

Overall, AD+LB+ consistently emerged as the most atrophic subgroup at baseline in the MTL and middle temporal regions and demonstrated the steepest declines in multiple cortical and subcortical structures. Whereas isolated LB often yields borderline or nonsignificant effects, its combination with AD markedly intensified neurodegeneration beyond single-pathology levels. Therefore, LB co-pathology compounds both baseline tissue loss and subsequent atrophy rates, aligning with the brain-age–based results in indicating a synergistic disruption of normal structure.

### 2.8. Longitudinal effect of co-pathology on cognition

**Extended Data Figure 4.**
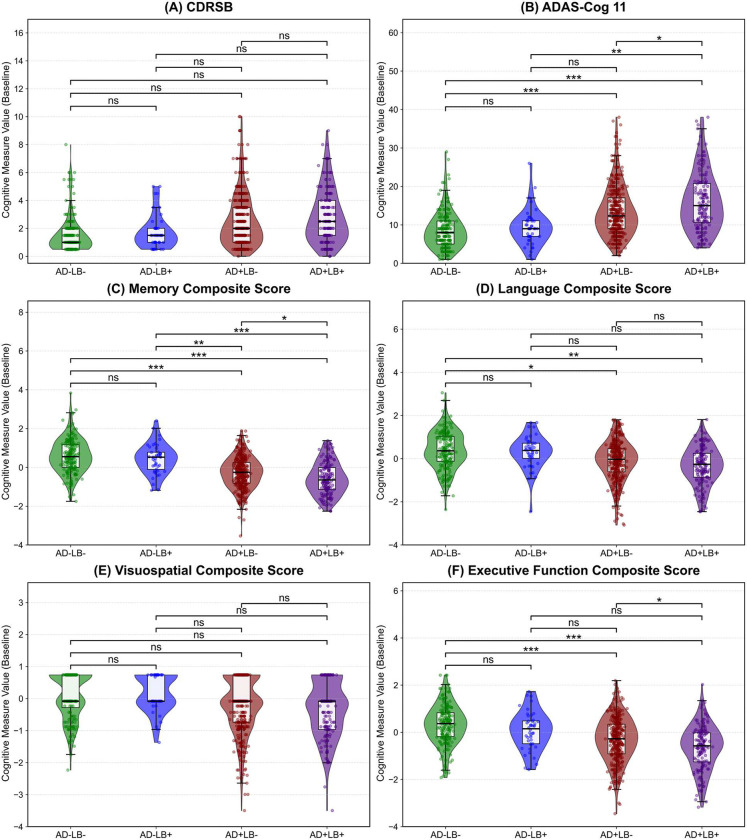
Baseline comparisons of global and domain-specific cognitive measures across AD/LB subgroups. **(A)** CDR Sum of Boxes (CDRSB) and **(B)** ADAS-Cog 11 (ADAS11) illustrate global cognitive measures; and **(C–F)** show the domain-specific performance: Memory, Language, Visuospatial, and Executive composites, respectively. Each violin plot depicts the distribution of the cognitive measure at baseline, with boxplots overlaid to illustrate medians and quartiles. The top bars and corresponding significance labels reflect pairwise group comparisons under a generalized linear model adjusted for baseline age, sex, cognitive state, and level of education, with p-values corrected for multiple comparisons. (*ns*: not significant; *p < 0.05; **p < 0.01; ***p < 0.001).

**Figure 7.**
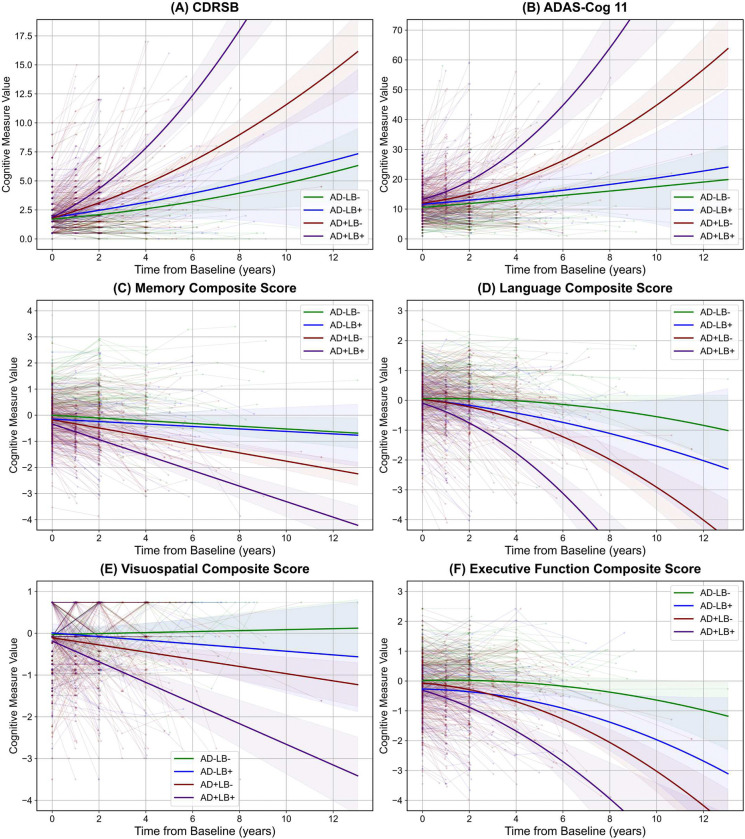
Longitudinal trajectories of global and domain-specific cognitive measures across AD/LB subgroups. **(A)** CDR Sum of Boxes (CDRSB) and **(B)** ADAS-Cog 11 (ADAS11) illustrate global cognitive decline over time; **(C–F)** show the domain-specific performance: Memory, Language, Visuospatial, and Executive composites, respectively. Thin lines represent each individual’s time course, whereas thicker lines and shaded areas show the estimated trajectories (linear or quadratic models are chosen based on the Bayesian Information Criterion (BIC)) and corresponding 95% confidence intervals. Notably, the co-pathology subgroup (AD+LB+) exhibits the steepest acceleration (for quadratic trajectories) or slope (for linear trajectories) of decline in both global and domain-specific outcomes. Specifically, *vs.* AD+LB−, AD+LB+ shows significantly greater worsening in CDRSB (*p* < 0.001), ADAS11 (*p* < 0.01), Memory (*p* < 0.01), Language (*p* < 0.01), Visuospatial (*p* < 0.001), and Executive function (*p* < 0.05). These findings indicate that LB co-pathology amplifies and hastens the deterioration already driven by AD-specific pathologies. All models are corrected for baseline age, sex, cognitive state, and level of education. Also, all *p*-values are adjusted for multiple comparison. AD: Alzheimer’s Disease; LB: Lewy Body; CDRSB: Clinical Dementia Rating Sum of Boxes; ADAS11: Alzheimer’s Disease Assessment Scale-Cognitive Subscale 11; BIC: Bayesian Information Criterion.

Building on our saliency map results, which identified key regions of atrophy and were further validated by our longitudinal analysis, we next explored the cognitive manifestations of these structural declines to determine whether LB co-pathology similarly accelerates cognitive deterioration over time. We conducted longitudinal mixed-effects analyses on two global cognition measures—Clinical dementia rating, sum of boxes (CDRSB) and Alzheimer’s disease assessment scale cognitive subscale (ADAS-Cog-11)—as well as four domain-specific cognitive composites (memory, language, visuospatial, and executive functioning). Model selection relied on BIC comparisons. For CDRSB and ADAS-Cog-11, a quadratic model was preferred. For domain-specific composites, a linear model was preferred for memory and visuospatial function, whereas language and executive function followed quadratic trajectories. Extended Data Figure 4 presents representative baseline comparisons, while Figure 7 depicts longitudinal trajectories across pathology subgroups, highlighting differential patterns of cognitive decline over time. Additional analyses for Mini-Mental State Examination (MMSE), modified Preclinical Alzheimer’s Cognitive Composite (PACC) variants, and Functional Activities Questionnaire (FAQ) are presented in the **Supplementary Figures 1, 2**.

At baseline, none of the pathology subgroups differed significantly from AD−LB− in CDRSB. However, in ADAS-Cog-11, AD+LB− (*β* = 1.42, *p* < 0.001) and AD+LB+ (*β* = 2.73, *p* < 0.0001) both exceeded AD−LB−, and pairwise contrasts revealed that AD+LB+ was also worse than AD−LB+ and AD+LB− (*p* < 0.01, < 0.05). Over time, AD+LB+ exhibited a significantly larger acceleration in both CDRSB and ADAS-Cog-11, relative to all other subgroups (e.g., *vs.* AD+LB−: *β* = 0.09, *p* < 0.001 for CDRSB, and *β* = 0.32, *p* < 0.01 for ADAS-Cog-11, and *vs.* AD−LB+: *β* = 0.12, *p* < 0.001 for CDRSB, and *β* = 0.52, *p* < 0.01 for ADAS-Cog-11).

Among the domain-specific composites, we observed a similar pattern of intensified decline in AD+LB+. Baseline comparisons revealed that memory and language displayed the most prominent baseline differences, with AD+LB− and AD+LB+ each significantly worse than AD−LB− (*p* < 0.0001) and AD+LB+ further below AD+LB− (*p* < 0.05) and AD−LB+ (*p* < 0.0001) in memory. Visuospatial showed milder baseline deficits, but AD+LB− and AD+LB+ were still lower than AD−LB− in executive function (*p* < 0.0001), whereas AD−LB+ showed no significant effects. In executive function, pairwise tests further revealed that AD+LB+ was lower than AD+LB− (*p* < 0.05) and AD−LB+ (*p* > 0.05). Over time, across all four composite scores, both AD+LB− and AD+LB+ declined significantly faster than AD−LB− (memory: *p* < 0.001 and *p* < 0.0001, language: *p* < 0.05 and < 0.01, visuospatial: *p* < 0.01 and < 0.0001, executive function: both *p* < 0.05), with AD+LB+ again showing the most pronounced declines (*vs.* AD+LB−: memory: *β* = −0.13, *p* < 0.01, language: *β* = −0.02, *p* < 0.01, visuospatial: *β* = −0.16, *p* < 0.001, executive function: *β* = −0.005, *p* < 0.05, and *vs.* AD−LB+: memory: *β* = −0.25, *p* < 0.0001, language: *β* = −0.04, *p* < 0.05, visuospatial: *β* = −0.20, *p* < 0.0001, executive function: *β* = −0.01, *p* < 0.05).

Overall, LB positivity in the absence of AD exerted limited influence, but in conjunction with AD, generated a pronounced intensification of cognitive deficits across global and domain-specific measures.

## 3. DISCUSSION

In this multimodal study, leveraging an interpretable DL model on structural MRI scans from a large, multi-cohort dataset, and incorporating a comprehensive set of cognitive measures along the recently developed CSF α-syn SAA results, we provided compelling evidence that individuals harboring concomitant AD and LB pathology (AD+LB+) exhibit markedly accelerated neurodegeneration, compared to isolated AD or LB. Specifically, our findings expand on prior reports suggesting that the prevalent co-occurrence of LB and AD-related pathologies exerts a synergistic effect, driving a significantly greater deviation in brain age gaps, more pronounced and extensive region-specific atrophy, and steeper cognitive decline compared to either pathology alone. Notably, these synergistic effects are pronounced across different key regions, including the MTL, occipital lobe, and NBM, suggesting an enhanced vulnerability of circuits typically involved in memory, attention, visuospatial and executive functioning.

The DL model, trained on a large, multi-cohort dataset of CU individuals demonstrated a remarkable ability to capture normative brain aging, with a high *R*^*2*^ and strong correlation reflecting its capacity to generalize across cohorts. Notably, the small average BAG (close to zero) further speaks to the reliability in modeling healthy brain aging. Based on this foundation and aligned with previous studies demonstrating the utility of BAG as a sensitive biomarker for neurodegeneration^26–28,36,52^, we assessed the deviations from these trajectories in CI individuals with AD/LB pathologies. Significant elevated brain age gaps were observed across all pathological subgroups, with the co-pathology subgroup showing the most elevated and rapidly rising brain age gaps over time, exceeding not just CU individuals, but also those with either AD or LB pathology alone. These findings reinforce and extend emerging evidence that LB pathology imposes an additional neurodegenerative burden on top of established AD processes^2,24^.

Sex-stratified analyses revealed intriguing differences in brain age gaps. In AD− subgroups (AD−LB− and AD−LB+), males had significantly higher brain age gaps than females; however, when AD pathology was present (AD+LB− and AD+LB+), the pattern reversed, with females showing more pronounced aging. These findings align with prior studies indicating that female brains often appear younger on MRI in the absence of AD but show accelerated aging once AD-specific processes are present^25^. Our results extend this viewpoint, suggesting that LB co-pathology can intensify such sex-specific vulnerability, potentially via interactions with hormonal (e.g., estrogen levels reduction during menopause) and genetic factors (e.g., APOE-ε4). Although future research is needed to dissect mechanistic underpinnings, our findings highlight the necessity of accounting for sex differences in AD/LB pathology studies.

The interpretability afforded by our DL-driven saliency maps provided a critical window into the specific brain regions most affected by co-pathology, guiding our investigation of corresponding region-specific atrophies and domain-specific cognitive decline.

Our analyses indicated that AD+LB+ shows an expanded set of salient regions, combining canonical AD targets (e.g., the MTL) with those frequently implicated in LB disease (e.g., the occipital cortex). Specifically, these areas exhibited greater saliency in driving the significantly higher brain age gaps observed in this subgroup, reflecting more pronounced atrophy compared to other subgroups. Longitudinal volumetric analyses further reinforced this finding, demonstrating that AD+LB+, relative to all other subgroups, experiences steeper volumetric atrophies in these key structures. A particularly striking example of this was observed in the MTL, where AD+LB+ exhibited a steeper decline in volume than AD+LB−, suggesting a potential synergy between α-synuclein and other AD-related processes in limbic regions. This accelerated MTL atrophy was paralleled by a steeper decline in memory performance over time in AD+LB+, implying a connection between structural degeneration in this critical memory circuit and its clinical manifestation. Beyond the MTL, our analyses revealed a more distributed pattern of vulnerability in AD+LB+, encompassing the occipital lobe, the posterior aspect of the middle temporal cortex, and basal ganglia. These regions also displayed heightened saliency in our DL model, accelerated atrophy in our longitudinal volumetric analyses, and were paralleled with declines in visuospatial, language, and executive function, respectively.

Prior neuropathology and MRI-based studies of LB pathology–associated atrophy have often implicated medial temporal structures, yet these findings have been inconsistent across cohorts and analytic methods^3,45–48^. Moreover, while Wisse et al.^3^ highlighted the NBM as a primary atrophic focus in LB-positive individuals, our DL-based saliency map analyses suggest that LB pathology extends beyond the NBM alone, encompassing parts of the MTL, the right cingulum and precuneus, and left fusiform gyrus, reinforcing the notion that LB pathology–related atrophy can reach multiple cortical and subcortical regions.

These interwoven findings from saliency maps, region-specific atrophy analyses, and longitudinal cognitive tracking illustrate a coherent portrait of synergistic co-pathology; LB pathology not only broadens the scope of structural decline beyond canonical AD patterns but also accelerates atrophy in core AD target areas. This multifaceted disruption manifests clinically as more pronounced and wide-ranging cognitive deficits.

The concept of accelerated brain aging (i.e., larger BAGs) has garnered attention as a framework for understanding how neurodegenerative diseases depart from normative aging. While prior studies^25,26,36^ have established BAG as a marker of neurodegeneration, they have largely relied on clinical diagnoses rather than direct biomarker evidence of pathology. By leveraging *in vivo* detection of misfolded α-synuclein alongside amyloid and tau markers, we provide critical evidence linking BAG to underlying LB pathology—especially in tandem with AD. To our knowledge, this is the first study to apply brain age modeling in the context of biomarker-confirmed AD/LB co-pathology, revealing its sensitivity to the compounded effects of α-synuclein and amyloid-tau pathologies. The ability of our interpretable DL model to capture deviations from normative aging trajectories provides a powerful framework for distinguishing pathology-driven neurodegeneration from typical senescence. Moreover, our longitudinal analyses established brain age gap as a dynamic marker capable of tracking disease progression and identifying individuals at heightened risk of rapid decline.

This study has certain limitations that warrant consideration. Although our cohorts were relatively large and well-characterized, the inclusion criteria for the ADNI cohort excluded individuals with overt parkinsonism or DLB. Therefore, the AD−LB+ subgroup is likely not representative of all individuals with primary synuclein pathology and the relatively small number of individuals in this subgroup limits the statistical power of findings relating to this subgroup. Future multi-cohort expansions are needed to ensure broader demographic and clinical representation, including individuals meeting diagnostic criteria for PD with or without cognitive impairment or DLB. The intricacies of how TDP-43 (a common co-pathology to AD and mixed AD/LB cases^53^), or vascular pathologies which might intersect with AD+LB+ also remain to be explored. Furthermore, although the concept of brain age gap is extensively used in the literature, and our DL model achieved high accuracies, BAG is still an indirect measure of neurodegeneration and may not capture all aspects of pathological brain aging. Additionally, while our DL model robustly captures normal aging trajectories, the interpretability of saliency maps requires careful consideration. Although saliency maps provide valuable insights into the regions driving brain age predictions, they do not directly represent the underlying biological mechanisms. Moreover, while we used MRI, a less invasive, more affordable, and safer imaging modality^54^ compared to other imaging techniques such as PET, future studies may extend our findings by incorporating additional neuroimaging methods, such as FDG-PET, diffusion-weighted MRI, or functional MRI, which can offer valuable complementary insights into the pathophysiology of AD/LB co-pathology, to provide a more comprehensive understanding of its neurobiological impact. Lastly, our longitudinal volumetric analysis focused on a subset of key regions, chosen from the full pool provided by the standard FreeSurfer *recon-all* function and the Desikan atlas. Future investigations should consider incorporating additional relevant regions defined by alternative brain atlases to achieve a more comprehensive characterization of AD/LB co-pathology–related neurodegeneration.

From a clinical and translational standpoint, our results underscore the importance of accurately identifying LB pathology in individuals with AD. Failure to consider LB status could overlook individuals at risk for more aggressive disease progression, potentially confounding clinical trial outcomes or real-world interventions. Emerging disease-modifying therapies targeting Aβ and tau may demonstrate reduced clinical efficacy if α-synuclein simultaneously drives neuronal loss and cognitive deterioration. Alternatively, understanding that LB pathology can intensify amyloid-tau pathology may spur interest in combination therapies tackling multiple proteinopathies at once. These insights may reshape how we diagnose, monitor, and treat individuals with coexistent proteinopathies, propelling research toward integrated therapeutic approaches that account for multiple neurodegenerative drivers.

## 4. METHODS

### 4.1. Study cohort

The data analyzed in this study were obtained from several established cohorts, each operating under its own ethical oversight and approved data acquisition protocols, where in each, all participants provided appropriate informed consent. All studies received approval from their respective institutional review boards or equivalent ethics committees. To capture normal aging trajectories using our brain age estimation model based on the MRI scans, we first selected cognitively unimpaired individuals across five major cohorts: ADNI^‡^ (*n* = 348; 71.3 ± 6.2 years, 65.0% female), AIBL^49^ (*n* = 359; 71.8 ± 6.1 years, 61.8% female), NACC^§^ (*n* = 2304; 68.6 ±10.7 years, 68.3% female), CamCAN^**^ (*n* = 623; 55.4 ± 17.9 years, 50.9% female)^50,51^, and HCP-A^††^ (*n* = 721; 60.2 ± 15.6 years, 56.2% female). To avoid data leakage and overfitting, only the first MRI scan per participant was included, yielding a total of 4,355 scans from individuals aged 23 to 100 years.

Given the longitudinal nature of ADNI, many cognitively unimpaired participants had serial scans acquired at different time points (on average, 5.0 ± 2.6 scans per participant). We excluded any scans that were used in the training phase from this cohort, and leveraged the remaining longitudinal scans from these cognitively unimpaired individuals in ADNI (*n* = 139, 691 scans; mean age 72.6 ± 5.9 years; 55.4% female) both to evaluate the model’s reliability in capturing normal aging trajectories within the same ADNI database—from which AD/LB pathology subgroups would later be derived—and to establish a reference point for subsequent comparisons with those pathological cohorts.

We next identified four cognitively impaired AD/LB pathology subgroups from the ADNI cohort. Participants included in these subgroups had their α-syn status determined via the most recent CSF α-syn SAA test, along with available data on CSF Aβ_42_ and p-tau_181_ within one year—biomarkers for Aβ and tau—and at least one T1-weighted MRI scan taken within one year of the SAA test. CSF concentrations of Aβ_42_ and p-tau_181_ were measured using the Elecsys CSF immunoassay^55^. AD-positivity was defined using the CSF p-tau_181_/Aβ_42_ ratio based on a previously specified threshold of 0.021 against [18F]florbetapir amyloid-PET, as recommended by the ADNI Biomarker Core Steering Committee (https://adni.loni.usc.edu/methods/). LB status was determined using the α-synuclein SAA test, performed at the Amprion Clinical Laboratory and validated for clinical use in accordance with Clinical Laboratory Improvement Amendment (CLIA) requirements^2^. Details of the methodology can be found in Arnold et al^22^. Based on the results of the p-tau_181_/Aβ_42_ ratio and SAA α-syn status, participants were classified into one of four AD/LB pathology subgroups: AD+LB+ (*n* = 166; 74.3 ± 7.2 years, 39.2% female), AD+LB− (*n* = 396; 73.5 ± 7.0 years, 42.4% female), AD−LB+ (*n* = 46; 73.8 ± 7.2 years, 28.3% female), and AD−LB− (*n* = 195; 72.0 ± 8.2 years, 40.5% female). Participants with intermediate SAA values were excluded. Individuals presenting with parkinsonism or other overt non-AD neurological disorders were excluded from ADNI, thereby, while this dataset is valuable for investigating AD/LB pathologies, it aims to exclude participants meeting clinical criteria for PD or DLB.

### 4.2. MRI data pre-processing and Quality Control Assessment

All raw T1-weighted MRI scans were processed using FreeSurfer’s recon-all pipeline (FreeSurfer v 7.1.1), which provides fully automated skull-stripping, motion correction, normalization of nonuniform signal intensities, and Talairach space transformation, as well as tissue segmentation and removal of nonbrain structures^56^. FreeSurfer was selected due to its established reliability in the literature, its comprehensive and reproducible workflow, and its capacity to facilitate analyses of neuroanatomical structures. Following recon-all, each MRI underwent an erosion–dilation step to refine tissue boundaries and was then affinely registered to the MNI152 atlas using Advanced Normalization Tools (ANTs) to achieve a consistent spatial framework for further analyses.

Ensuring the quality of the processed MRI data was critical, as the presence of low-quality scans can bias model training and evaluation. Although Qoala-T^57^ was used as an initial automated quality control tool, we complemented it with additional outlier detection methods to identify images that were not appropriately processed by FreeSurfer. These measures were implemented both before and after data normalization to capture a wide range of intensity- and structure-related anomalies. Prior to normalization, we applied intensity-based criteria to detect scans exhibiting extreme deviations in global brightness, contrast, and overall intensity distribution. For instance, we identified scans whose mean or standard deviation of voxel intensities substantially diverged from the dataset norm, as well as those whose voxel intensities, when standardized, fell significantly outside expected ranges. Such deviations often suggest technical issues or scanner-specific artifacts that warrant exclusion or closer inspection. Following normalization, we shifted our focus toward detecting more subtle anomalies that would be less apparent in raw intensity space. We used principal component analysis (PCA) to represent each scan in a lower-dimensional space capturing the major patterns of variability, and then measured each scan’s distance from the multivariate center using the Mahalanobis metric. This approach facilitated the identification of images that, despite appearing normalized, still exhibited atypical structural or intensity profiles. In addition, we applied an Isolation Forest algorithm, which relies on iterative feature splitting, to identify scans that could be more easily isolated from the overall distribution, thus flagging subtle irregularities not explained by global intensity measures or principal components alone.

By integrating these checks before and after normalization, we established a multi-layered quality control strategy. Notably, each outlier detection method was applied separately to each subgroup, rather than to the entire pooled dataset, to ensure that flagged scans represented true anomalies relative to their own clinical context. Following each round of outlier detection, all identified images were manually inspected to confirm that they were genuine outliers rather than artifacts of the detection process. This careful, stepwise approach ensured that only high-quality, representative MRI data informed the subsequent stages of deep learning model training and validation, ultimately enhancing the robustness and interpretability of our results.

### 4.3. Deep learning model architecture and training

A 3D-DenseNet architecture^58^ was chosen to predict brain age from the processed T1-weighted MRI scans of cognitively unimpaired individuals, primarily due to its proven performance in handling such data^26^. As depicted in Figure 1, the network comprises four dense blocks containing 3, 6, 12, and 8 layers, respectively, with each block separated by a transition block. Within each dense layer, a 1×1×1 bottleneck convolution is set to scale the current in-channel dimension, followed by a 3×3×3 convolution. The initial convolution layer uses a 5×5×5 kernel, and after the final dense block, a 3×3×3 convolution is applied before a global average pooling layer. The resulting features are fed into a fully connected layer predicting a single scalar, called the brain age. This architecture contains 251,098,737 trainable parameters. Although 3D-DenseNet architectures often rely on a smaller bottleneck dimension^26^, we opted for a configuration in which we allow the 1×1×1 convolution to scale with the current in-channel dimension, rather than a fixed multiple of the growth rate. This larger design was motivated by our diverse, multi-cohort dataset, where we found that the additional capacity helped capture subtle inter-subject variations and group differences without overfitting, as evidenced by steadily improving validation losses and final test MAE.

Training proceeded in a staged manner, with five iterative cycles of 15 epochs each. Adam was chosen as the optimizer, and early stopping was applied to halt training if the validation loss did not improve for several epochs (patience=6), ensuring that the model did not overfit. Extensive hyperparameter tuning was conducted to identify the optimal configuration for batch size, kernel sizes, growth rates, loss function, and learning rate, while the optimizer and the early stopping callback were kept constant throughout the tuning phase, providing a controlled environment to evaluate each combination. After systematically evaluating multiple configurations, we selected the best-performing hyperparameters based on their validation set performance. The final choice included a mini-batch size of 8, a mean absolute error (MAE) loss function for directly quantifying prediction errors in years, and an initial learning rate of 5*e*^−6^. Whenever the validation performance plateaued over all epochs of an iteration, we reduced the learning rate by a factor of 0.7 to facilitate convergence toward an optimal solution. Random seeds were fixed at 42 to enhance reproducibility, and during training, the best model state was periodically saved whenever validation performance improved, ensuring that we retained the optimal set of parameters for downstream evaluation. All training was performed in PyTorch using two A100 GPUs in parallel, allowing efficient handling of the large 3D volumes and substantial model complexity.

To ensure robust estimation of generalizable brain aging patterns, we assembled a cohort of 4,355 cognitively unimpaired participants, aged 23–100 years. To prevent data leakage and to rigorously assess generalizability, we split the dataset into training (80%, *n* = 3,484; 65.8 ± 13.5 years, 63.6%), validation (10%, *n* = 435; 66.3 ± 12.7 years, 61.4% female), and test (10%, *n* = 436; 66.2 ± 12.9 years, 61.7% female) sets. The splits were performed using stratified sampling based on discretized age bins, ensuring a balanced age distribution across subsets. We also checked that sex distribution did not differ significantly among the training, validation, and test sets, preserving comparability for subsequent analyses, including those involving sex effects. As mentioned before, we only included the first available MRI scan per participant, preventing any subject-level overlap between the training, validation, and testing phases. By integrating a robust 3D-DenseNet model, rigorously stratified data splitting, careful hyperparameter tuning, and adaptive learning rate and early stopping strategies, we aimed to achieve a stable, generalizable brain age estimation pipeline. This carefully engineered training process ensures that the final model reflects genuine aging patterns rather than artifacts of sampling, preprocessing, or overfitting.

### 4.4. Saliency maps analysis

We employed a gradient-based saliency mapping approach to visualize the spatial distribution of features that most strongly influenced the predictions of our 3D-DenseNet model. Following the extension of previously established 2D saliency techniques^59^ to the 3D domain^25^, each input image (volume $V_{0}$) was passed through the trained 3D-DenseNet model with a score function S(V) and voxels in $V_{0}$ are ranked based on their importance to the score function. Although this score function is inherently nonlinear with respect to V, a local linear approximation around $V_{0}$ can be obtained using a first-order Taylor expansion. In this approximation, voxel importance is inferred from the partial derivatives of S(V) with respect to the input V at $V_{0}$, reflecting how infinitesimal changes in voxel intensity affect the predicted brain age.

More specifically, the procedure for generating saliency maps was as follows. First, we fed each subject’s MRI volume into the 3D-DenseNet in evaluation mode in PyTorch, identified the output neuron with the highest contribution to the predicted brain age, and then computed the gradients of that top-scoring output with respect to the input volume. These gradients capture how sensitively the model’s prediction responds to each voxel’s intensity. To further enhance interpretability and mitigate noise, we applied Gaussian smoothing to the saliency maps. This additional step provided clearer, more coherent patterns of feature relevance across the 3D volume. Smoothed saliency maps were generated for all cognitively unimpaired individuals as well as for all AD/LB participants, enabling a nuanced examination of how structural brain features differentially influenced the deep learning model’s brain age estimates.

Subsequently, we averaged individual saliency maps within each diagnostic group to obtain representative mean saliency patterns. To elucidate differences in salient patterns across groups, we computed difference maps by subtracting the averaged saliency maps of one cohort from another. These difference maps highlight the regions in the brain MRI volumes that differentially influence the model’s prediction across distinct clinical conditions. All surfaces were visualized using the BrainNet Viewer^60^.

### 4.5. Neuroanatomical assessments

To examine the impact of co-pathology on regional brain atrophy, we used the volumetric measures extracted from FreeSurfer’s *recon-all* pipeline. For cortical parcellations, we used the Desikan–Killiany atlas and for subcortical segmentation, we used the FreeSurfer automated segmentation (aseg). We focused on four major anatomical compartments that are commonly implicated in AD and/or LB pathology. Specifically, the medial temporal lobe (MTL) included the bilateral hippocampus, entorhinal cortex, amygdala, and parahippocampal gyrus; the basal ganglia comprised the bilateral caudate, putamen, accumbens, and pallidum; the occipital lobe encompassed the lateral occipital, cuneus, pericalcarine, and lingual cortices; and the middle temporal region consisted of the mid-portion of the bilateral temporal cortex. We also considered fusiform, cingulum and insual in our analysis. All volumes were normalized to each individual’s intracranial volume (ICV) to account for differences in head size. These regions were chosen based on their established vulnerability in AD and LB disorders, thereby capturing the key neuroanatomical targets of both pathologies.

### 4.6. Neuropsychological assessments

For the cognitive performance evaluation among the AD/LB pathology groups, we considered a comprehensive set of neuropsychological test scores that were completed within one year of SAA, including measures of global cognition such as the Mini-Mental State Examination (MMSE) and Clinical Dementia Rating Sum of Boxes (CDR-SB), as well as general cognition and functional measures like the Alzheimer’s Disease Assessment Scale-Cognitive Subscale (ADAS-Cog-11), Functional Activities Questionnaire (FAQ), and the modified Preclinical Alzheimer’s Cognitive Composite (mPACCdigit and mPACCtrailsB). Cognitive domain scores were also evaluated, reflecting memory, language, visuospatial function, and executive function. These scores provided a robust framework to assess cognitive trajectories and differences across pathological groups.

### 4.7. Statistical analysis

We first assessed group-level demographic differences among the four pathology subgroups (AD−LB−, AD+LB−, AD−LB+, and AD+LB+) using chi-square tests for categorical variables and one-way analysis of variance (ANOVA) for continuous variables. Similarly, before training the brain age deep learning model, we used a one-way ANOVA and a chi-square test, respectively, to ensure there were no significant differences in chronological age and sex distribution across the training, validation, and test subsets of cognitively unimpaired individuals. To assess the accuracy of our brain age estimations from the MRI scans, we computed the mean absolute error (MAE) between the predicted brain age (BA) and chronological age (CA), along with Spearman’s correlation (r), and the coefficient of determination ($R^{2}$). The Spearman’s correlation coefficient evaluated the monotonic relationship between BA and CA, while $R^{2}$ reflected the proportion of the variance in BA explained by CA in a linear model.

The brain age gap (BAG)—the difference between a deep learning–predicted brain age based on the T1-weighted MRI scan and a participant’s chronological age (BA - CA)—is used to assess the neurodegenerative burden. To account for the well-known bias in brain age predictions, where brain age gaps are systematically correlated with chronological age—resulting in overestimation in younger individuals and underestimation in older individuals due to regression dilution^61,62^—we applied a modified linear bias correction method described by Smith et al^63^. Specifically, we fitted a linear regression BA=a×CA+b to the validation data of the cognitively unimpaired group and subsequently computed the corrected brain age gap as (BA-b)/a-CA. The same a and b coefficients derived from the validation set of the cognitively unimpaired individuals were applied to the test set of this group, the cognitively unimpaired longitudinal subset from ADNI, and the four pathology subgroups for bias correction.

We compared the corrected brain age gaps among all groups, as well as within each sex across groups, using one-way ANOVA followed by Holm-Šídák post hoc tests. To further assess sex-related differences within each group, we conducted two-sample *t*-tests comparing females and males, again applying Holm-Šídák correction to mitigate type I error inflation due to multiple testing. In all analyses, chronological age was included as a nuisance covariate to control for its potential confounding effects.

To investigate group-specific trajectories of brain aging, structural atrophy, and cognitive function over time, we employed linear mixed models (LMMs) with random intercepts and slopes, while all baseline group differences for each outcome were examined using a general linear model. The linear mixed models included the interaction between the pathology group and time as a predictor to assess group-dependent rates of progression. Additionally, we incorporated a quadratic interaction term for group by time squared (time^2^) to account for potential non-linear changes. Model preference between linear and quadratic models was determined using the Bayesian Information Criterion (BIC)^64^, where the model with the lower BIC value was selected for its balance between goodness-of-fit and parsimony. A significant group-by-time interaction term was interpreted as evidence of group-specific differences in the rate of brain aging-related or cognitive functioning-related changes over time, while a significant group-by-time^2^ interaction term suggested non-linear (i.e., accelerated) changes. All models were corrected for baseline age, sex, cognitive state^‡‡^, and level of education. Pairwise group comparisons were corrected for multiple testing using FDR correction method to ensure the robustness of our findings. All statistical analyses were conducted in Python, using the scikit-learn, Statsmodels and SciPy libraries. Statistical significance was set at two-sided (*p* < 0.05).

## Extended Data

## Supplementary Material

Supplementary Files

This is a list of supplementary files associated with this preprint. Click to download.

• CoPathologyManuscript20250604SupplementaryMaterials.pdf

## Figures and Tables

**Table 1.** Baseline demographic, cognitive, and volumetric characteristics of cognitively impaired individuals by AD/LB biomarker status. Participants were drawn from the ADNI cohort and classified into four subgroups according to their corresponding CSF biomarker results: AD-LB- (*n* = 195), AD-LB+ (*n* = 46), AD+LB- (*n* = 396), and AD+LB+ (*n* = 166). Values are presented as mean ± s.d. or counts (%). Cognitive measures include MMSE (Mini-Mental State Examination), CDR-SB (Clinical Dementia Rating Sum of Boxes), ADAS11 (Alzheimer’s Disease Assessment Scale-Cognitive Subscale, 11-item), domain-specific composites (Memory, Language, Visuospatial, Executive), Functional Activities Questionnaire (FAQ), and modified Preclinical Alzheimer’s Cognitive Composite metrics (mPACCdigit, mPACCtrailsB). Volumetric measures (normalized to intracranial volume) cover medial temporal, basal ganglia, occipital, and related regions often affected by AD and/or LB pathology. These baseline data show that participants with copathology (AD+LB+) tend to have more cognitive impairments and smaller volumetric measures.

	AD-LB- (*n* = 195)	AD-LB+ (*n* = 46)	AD+LB- (*n* = 396)	AD+LB+ (*n* = 166)
**Demographics**				
Age [year]	72.0 ± 8.2	73.8 ± 7.2	73.5 ± 7.0	74.3 ± 7.2
Sex, F (%)	79 (40.5%)	13 (28.3%)	168 (42.4%)	65 (39.2%)
Education [year]	16.2 ± 2.6	16.8 ± 2.4	15.9 ± 2.7	15.9 ± 3.0
**Cognitive Status**				
MCI	177	40	235	79
Dementia	18	6	161	87
**APOE-e4**				
Non-carrier	152	40	135	49
Heterozygous, 1	42	3	196	81
Homozygous, 2	1	3	65	36
**Cognition**				
MMSE	28.12 ± 1.97	27.74 ± 1.87	26.48 ± 2.64	25.58 ± 2.74
CDRSB	1.44 ± 1.28	1.29 ± 0.95	2.28 ± 1.81	2.67 ± 1.88
ADAS11	8.49 ± 4.65	9.08 ± 4.18	12.83 ± 6.44	15.45 ± 7.45
*Missing*	0	0	0	1
Memory	0.56 ± 0.97	0.44 ± 0.75	−0.34 ± 0.97	−0.74 ± 0.97
Language	0.38 ± 0.91	0.32 ± 0.76	−0.18 ± 1.04	−0.57 ± 1.05
Visuospatial	0.12 ± 0.68	0.19 ± 0.57	−0.23 ± 0.89	−0.48 ± 0.96
Executive function	0.4 ± 0.85	0.066 ± 0.79	−0.34 ± 1.05	−0.83 ± 1.08
FAQ	2.95 ± 5.51	4.33 ± 5.63	7.74 ± 7.68	9.96 ± 8.38
*Missing*	3	0	3	1
mPACCtrailsB	−3.69 ± 4.77	−4.66 ± 3.97	−9.49 ± 6.45	−12.12 ± 6.50
mPACCdigit	−4.23 ± 5.38	−4.89 ± 4.33	−10.18 ± 6.83	−12.65 ± 6.74
**Gray Matter Volumetrics (cm^3^)**				
Hippocampus	4.847 ± 0.937	4.540 ± 0.889	4.328 ± 0.792	4.089 ± 0.698
Parahippocampus	2.503 ± 0.498	2.432 ± 0.464	2.341 ± 0.435	2.227 ± 0.429
Entorhinal	2.334 ± 0.613	2.227 ± 0.518	2.106 ± 0.546	1.919 ± 0.523
Amygdala	1.997 ± 0.439	1.869 ± 0.427	1.712 ± 0.405	1.548 ± 0.363
Occipital lobe	28.397 ± 3.623	27.304 ± 4.063	26.913 ± 3.663	26.279 ± 3.330
Basal Ganglia	12.647 ± 1.658	12.238 ± 1.517	12.323 ± 1.555	12.028 ± 1.399
Middle Temporal	13.421 ± 1.849	12.744 ± 2.081	12.257 ± 2.063	11.688 ± 1.923
Cingulum	11.725 ± 1.377	11.386 ± 1.399	11.184 ± 1.441	10.884 ± 1.281
Insula	8.605 ± 0.961	8.417 ± 1.043	8.402 ± 0.966	8.151 ± 0.938
Fusiform	11.4 ± 1.459	11.279 ± 1.697	10.622 ± 1.623	10.366 ± 1.612

## Data Availability

All data used in this study are publicly available from the sources identified in the paper.

## Footnotes

‡ The ADNI (adni.loni.usc.edu) was launched in 2003 as a public-private partnership, led by Principal Investigator Michael W. Weiner, MD. The primary goal of ADNI has been to test whether serial magnetic resonance imaging (MRI), positron emission tomography (PET), other biological markers, and clinical and neuropsychological assessment can be combined to measure the progression of mild cognitive impairment (MCI) and early Alzheimer’s disease (AD). For up-to-date information, see www.adni-info.org.

§ Obtained from the NACC database (available at [https://naccdata.org]).

** Obtained from the CamCAN repository (available at http://www.mrc-cbu.cam.ac.uk/datasets/camcan/),

†† Provided by the Human Connectome Project in Aging (HCP-Aging), accessible at [https://www.humanconnectome.org/study/hcp-lifespan-aging].

‡‡ In ADNI, cognitive state is defined based on the Clinical Dementia Rating (CDR) global score, Mini-Mental State Exam (MMSE), and Wechsler Logical Memory II sub-scale, in line with ADNI’s screening and diagnostic criteria. More details are provided in the ADNI procedures manuals.

## Abbreviation Definition

AAL: Automated Anatomical Labeling
AD: Alzheimer’s disease
ADAS-Cog-11: Alzheimer’s disease assessment scale - cognitive subscale (11-item version)
ADNI: Alzheimer’s Disease Neuroimaging Initiative
AIBL: Australian Imaging, Biomarkers & Lifestyle
ANOVA: Analysis of variance
ANTs: Advanced normalization tools
Aβ: Amyloid beta
BA: Brain age
BAG (BA-CA): Brain age gap: the difference between the predicted brain age (via deep learning model) and chronological age, used as a proxy for neurodegeneration
BIC: Bayesian information criterion
CA: Chronological age
CamCAN: Cambridge Centre for Ageing and Neuroscience
CDRSB: Clinical dementia rating, sum of boxes
CSF: Cerebrospinal fluid
DL: Deep learning
DLB: Dementia with Lewy bodies
FAQ: Functional activities questionnaire
HCP-A: Human Connectome Project in Aging
ICV: Intracranial volume
LB: Lewy body
LMM: Linear mixed model
MAE: Mean absolute error
MCI: Mild cognitive impairment
MMSE: Mini-mental state examination
MRI: Magnetic resonance imaging
MTL: Medial temporal lobe
NACC: National Alzheimer’s Coordinating Center
NBM: Nucleus basalis of Meynert
PACC: Preclinical Alzheimer’s cognitive composite
PD: Parkinson’s disease
r: Spearman’s correlation
R2: Coefficient of determination
SAA: Seed amplification assay
